# Tracheal Resection for Critical Airway Obstruction in Morquio A Syndrome

**DOI:** 10.1155/2023/7976780

**Published:** 2023-05-03

**Authors:** Claire Frauenfelder, Elizabeth Maughan, Johnny Kenth, Reema Nandi, Simon Jones, Robert Walker, Bill Walsh, Nagarajan Muthialu, Iain Bruce, Richard Hewitt, Colin Butler

**Affiliations:** ^1^Great Ormond Street Hospital for Children NHS Foundation Trust, London, UK; ^2^University of Adelaide, Adelaide, Australia; ^3^UCL Great Ormond Street Institute of Child Health, Great Ormond Street Hospital for Children, London, UK; ^4^Department of Paediatric Anaesthesia, Royal Manchester Children's Hospital, Manchester University NHS Foundation Trust, Manchester, UK; ^5^The University of Manchester, The Faculty of Biology, Medicine and Health, Manchester, UK; ^6^The Willink Metabolic Unit, Manchester Centre for Genomic Medicine, Manchester University NHS Foundation Trust, Manchester, UK; ^7^Paediatric ENT Department, Royal Manchester Children's Hospital, Manchester University NHS Foundation Trust, Manchester Academic Health Science Centre, Manchester, UK; ^8^Divisions of Infection, Immunity and Respiratory Medicine, Faculty of Biology, Medicine and Health, University of Manchester, Manchester, UK

## Abstract

**Introduction:**

The primary cause of death in Morquio A syndrome (mucopolysaccharidosis (MPS) IVA) is airway obstruction, brought about by an inexorable and pathognomonic multilevel airway tortuosity, buckling, and obstruction. The relative pathophysiological contributions of an inherent cartilage processing defect versus a mismatch in longitudinal growth between the trachea and the thoracic cage are currently a subject of debate. Enzyme replacement therapy (ERT) and multidisciplinary management continue to improve life expectancy for Morquio A patients by slowing many of the multisystem pathological consequences of the disease but are not as effective at reversing established pathology. An urgent need has developed to consider alternatives to palliation of progressive tracheal obstruction to preserve and maintain these patients' hard-won good quality of life, as well as to facilitate spinal and other required surgery. *Case Report*. Following multidisciplinary discussion, transcervical tracheal resection with limited manubriectomy was successfully performed, without the need for cardiopulmonary bypass, in an adolescent male on ERT with the severe airway manifestations of Morquio A syndrome. His trachea was found to be under significant compressive forces at surgery. On histology, chondrocyte lacunae appeared enlarged, but intracellular lysosomal staining and extracellular glycosaminoglycan staining was comparable to control trachea. At 12 months, this has resulted in a significant improvement in respiratory and functional status, with corresponding enhancement to his quality of life.

**Conclusion:**

This addressing of tracheal/thoracic cage dimension mismatch represents a novel surgical treatment approach to an existing clinical paradigm and may be useful for other carefully selected individuals with MPS IVA. Further work is needed to better understand the role and optimal timing of tracheal resection within this patient cohort so as to individually balance considerable surgical and anaesthetic risks against the potential symptomatic and life expectancy benefits.

## 1. Introduction

Morquio A syndrome (mucopolysaccharidosis IVA) is a rare (1 in 400,000 live births) autosomal recessive lysosomal storage disease caused by an aberration or total deficiency of the enzyme N-acetylgalactosamine-6-sulphate sulfatase (GALNS). This deficiency leads to the accumulation of partially degraded metabolites of two ubiquitous glycosaminoglycans (GAGs: chondroitin-6-sulphate (C6S) and keratan sulphate (KS)) within lysosomes and the extracellular matrix (in comparison to Morquio B syndrome, caused by lack of *β*-galactosidase, where only KS accumulates). The ubiquitous nature of these proteins means that many organ systems are generally affected [1]. There is often considerable variability in phenotypical severity, but in most cases, growth ceases by eight years, with children becoming wheelchair-bound and sadly succumbing to the disease in their second decade.

Thanks to enzyme replacement therapy (ERT), individuals with Morquio A syndrome are living longer after earlier initial diagnosis and treatment [2]. The benefits of ERT on skeletal dysplasia progression are less clear-cut than those seen in the viscera [3, 4], but the ability to carry out orthopaedic interventions may be limited by the presence of airway and spinal cord compromise. Tortuous distortion and compression of the trachea advances over time, and patients experience worsening dyspnoea and opisthotonos (severe cervical hyperextension), as well as progressive vertebral abnormalities and instability, and development of spinal cord compression—this combination of issues particularly impacts quality of life and remains the leading cause of death for patients with Morquio A syndrome [5].

We report the novel and successful application of transcervical tracheal resection and limited manubriectomy as a surgical technique for severe airway obstruction in Morquio A syndrome, performed without the need for cardiopulmonary bypass.

## 2. Case Presentation

### 2.1. Patient Presentation

A 17-year-old male patient with Morquio A syndrome, under the long-term care of the inherited metabolic disorders (IMD) service at Manchester University NHS Foundation Trust (MFT), was referred to the Manchester Paediatric Otolaryngology (ENT) service in November 2019 for airway assessment due to progressive dyspnoea and opisthotonos. At the time of referral, the patient had received ERT for 10 years, having commenced treatment aged 78 months, and his quality of life was otherwise good, attending college, studying drama, and relying only on nocturnal BiPAP for respiratory support. Progressive fatigue due to increasing work of breathing over time left the patient unable to sustain a full school timetable, increased his reliance on powered mobility aids especially outside of home, and limited his sentences to 1 to 2 words.

**Figure 1:**
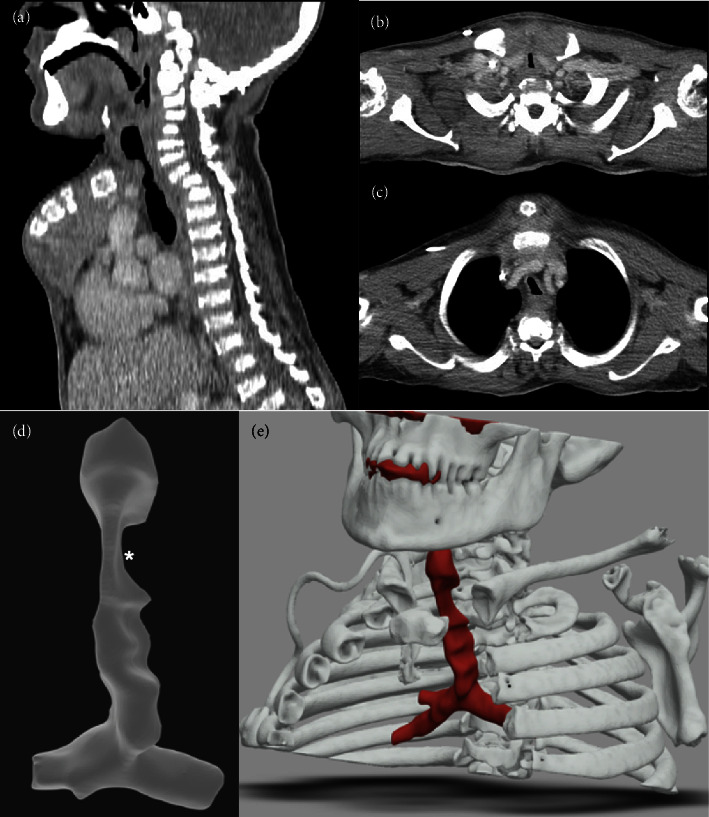
CT angiogram sagittal and (b) axial views immediately above and (c) below the manubrial head demonstrate multifactorial airway obstruction. Abnormal manubrium orientation and interposed brachiocephalic vasculature compressing the midtrachea into the c-spine, tracheal tortuosity, and tracheal ring deformity; shortened thorax with upper mediastinal compression; and cervico-thoracic kyphoscoliosis. (d-e) 3D-reconstructed images clearly demonstrate a tight thoracic inlet narrowing (^*∗*^).

Postpubertal CT imaging demonstrated characteristically tortuous multilevel airway obstruction with a focal segment of severe collapse at the level of the thoracic inlet (Figure 1). Airway assessment by endoscopic microlaryngobronchoscopy under general anaesthetic demonstrated a relatively normal pharyngeal, glottic, and subglottic airway with severe tortuosity of the proximal to middle trachea with a particularly tortuous stenosis beginning 36 mm beyond the glottis (Supplementary Video 1).

After multidisciplinary discussion involving local ENT, respiratory, physiotherapy, and IMD services, robust consensus was reached to pursue active treatment options for the patient rather than palliation, and the UK's National Tracheal service at GOSH was contacted regarding possible tracheal resection or airway stenting. Airway stenting was felt not to provide enough radial force to alleviate his airway stenosis and risked tracheal wall erosion by the stent. Localised resection of tracheal tortuosity at the thoracic inlet was felt likely to provide sufficient symptom relief without the need for cardiopulmonary bypass.

Several challenging factors needed consideration, and a series of collaborative multidisciplinary meetings were undertaken with the patient and his parents. These factors were as follows: the highly technical and challenging nature of the procedure, the patient's extremely high-risk spinal and respiratory comorbidities, the “*n*-of-one” nature of the procedure which has not previously been described in literature, and finally, the unpredictability of how underlying Morquio A disease might affect wound healing, with risk of surgical dehiscence or anastomosis failure. Further advice and input were also sought from the GOSH Clinical Ethics Committee, where an opinion in favour of proceeding with the planned procedure was reached [6] in June 2020.

### 2.2. Surgical Procedure

**Figure 2:**
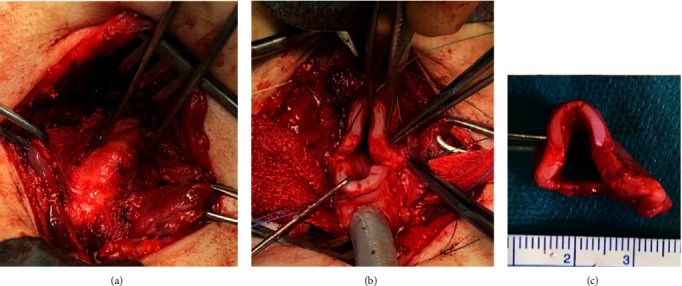
Clinical photographs at cricotracheal resection: (a) tracheal exposure via limited manubriectomy showed a first bend 36 mm distal to the glottis, leading into a 16 mm tortuosity terminating 28 mm above the carina. (b) The deformed tracheal segment, which had concertinaed leading to omega-shaped tracheal ring buckling, was resected without (c) cardiopulmonary bypass,

Transcervical surgical resection of the most affected proximal tracheal segment was performed in July 2020 (Figure 2). A midline, low cervical incision was made, and soft tissue elevated to expose the trachea, manubrium, and clavicular heads. A limited manubriectomy was performed after mobilisation of the ventral adherent soft tissue both for access and to remove extrinsic compression. The pretracheal tissue was reflected, the tortuous trachea exposed, and a temporary tracheostomy used to place a cuffed endotracheal tube (ETT) in the distal trachea. The airway was opened horizontally, and the deformed, tortuous proximal tracheal segment was inspected. There was an omega-shaped deformity of the tracheal cartilage, and the trachea itself was compressed in a concertina fashion, easily able to stretch and extend unlike normal trachea. A 16 mm tracheal segment was excised, involving 5 cartilaginous rings. Trachealis, the posterior trachea and its mucosa were primarily anastomosed, with heavy PDS sutures used to secure the cartilage rings laterally. The patient was reintubated transnasally, and the anterior tracheal rings reopposed using heavy PDS. The soft tissue was closed in multiple layers, and a Yeates drain was secured as an air-drain.

Given the need for prolonged neck extension with the incumbent risk of spinal cord compression throughout the procedure, spinal neuro-monitoring with motor and sensory evoked potentials was performed at the start of the procedure to determine baseline readings without neck extension as well as the maximal neck extension available without spinal cord compression, and this was actively rechecked throughout the case.

**Figure 3:**
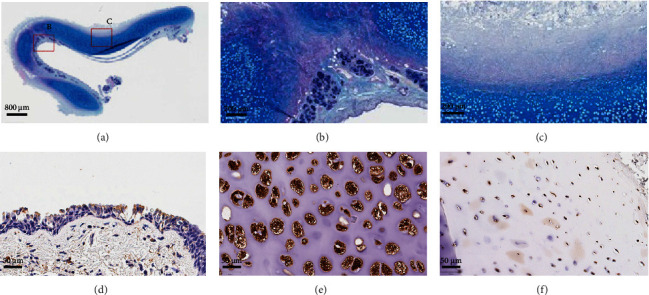
Histopathological characterisation of resected trachea: periodic acid Schiff staining showed comparable GAG staining to controls in (a) mucosa, (b) cartilage, and (c) pericartilagenous junctions. Lysosomal LAMP1 staining showed no additional staining intensity in (d) epithelium or (e) chondrocytes, however, (f) chondrocyte lacunae were markedly larger in diameter.

The resected tracheal segment was analysed by histology and immunohistochemistry for CD68 (macrophage marker) and LAMP1 (lysosomal associated membrane protein 1, overabundant in lysosomal storage disorders) (Figure 3). GAG deposition was not increased in the cartilage, perichondral zones, or submucosa. There was an increase in perichondral macrophage infiltration in comparison to normal trachea, but no evidence of foamy macrophage phenotypes or frank cartilaginous infiltration was found. Chondrocyte cell lacunae appeared markedly enlarged compared to normal tracheal cartilage, but LAMP1 staining intensity within the lacunae was comparable to normal trachea.

### 2.3. Postoperative Course

The patient was transferred to the paediatric intensive care intubated and ventilated. On the first postoperative day (POD), he developed bilateral pneumothoraces and chest drains were sited. Over subsequent days, sedation was gradually weaned, although the teenage patient found the difficulties associated with an inability to communicate extremely distressing; multidisciplinary input from physiotherapy and psychology were instrumental in his immediate postoperative recovery. He was successfully extubated on POD 4 onto noninvasive ventilatory support (NIV) as a bridging therapy.

It is our practice as the National Tracheal Service to use early postoperative endoscopic microlaryngobronchoscopy rather than CT as a measure of postsurgical change, as it is more clinically relevant with regards to the degree of luminal obstruction, enables interventional capability in the case of postsurgical granulations or insertion of luminal stents, and does not confer any additional radiation load. This was performed on POD 7 confirming that the tracheal anastomosis was healing well with no dehiscence or granulation formation (Supplementary Video 2). He was discharged home on POD 17, having weaned over 2 weeks to his preoperative nocturnal BiPAP settings.

**Figure 4:**
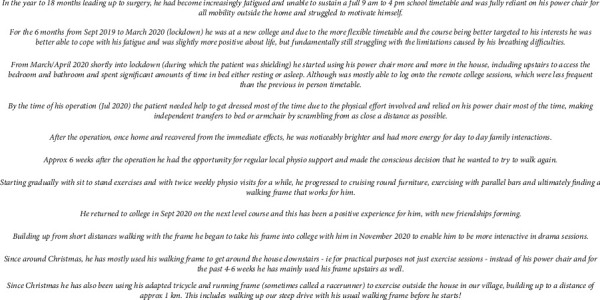
Narrative account by parents articulating the changes in quality of life after the procedure. We note that whilst the account is presented verbatim in the parents' own words, all identifying information has been removed.

Almost immediately after returning home, the patient found his work of breathing and energy levels were markedly improved. Over the course of the next 12 months, he has returned to exercise using his adapted tricycle and running frame and has returned to full-time college. He has shown extremely positive scores in all domains of the Glasgow Benefit Inventory (GBI) quality of life score (Figure 4 and Supplementary material available (here)).

## 3. Discussion

This case illustrates some new key concepts in the pathophysiology and management of Morquio A syndrome airway manifestations. It is generally thought that Morquio A syndrome's pathognomonic tracheal defects arise from (1) mediastinal and thoracic inlet crowding due to the brachiocephalic artery and the manubrium and (2) a longitudinal size mismatch of the trachea [7]. Others have noted a characteristic laxity of the tracheal tissue [7], and autopsy results from affected patients have shown an accumulation of GAGs within the tracheal wall coupled with marked macrophage infiltration [8, 9]. It was unclear which of these factors represented the dominant contribution to the airway defects in our patient, and longitudinal studies suggest that severely affected individuals continue to deteriorate despite ERT [10], often progressing to critical airway obstruction necessitating palliation with NIV despite having finished growing in puberty [11].

In our National Morquio A cohort, we have found no specific risk factor for the development of tracheal narrowing in some patients with MPS IVA and not others, and indeed one sibling with Morquio A may be affected to a far greater extent than another. The main indication for surgical intervention is based on a life-limiting respiratory obstruction alongside objective clinical markers (LFTs, CT, and MLB) compatible with tracheal narrowing as the primary cause. Patients all go through individualised MDT discussion (which fully include the patient and their family) as to whether their symptoms warrant the risk of surgical intervention.

We found that this patient's tracheal rings were under significant compressive forces, in contrast to a normal airway where the proximal and distal portions of the airway separate under tension when transected, which rendered a primary tension-free anastomosis straightforward. On histology, CD68 staining for macrophages did not show the foam cell phenotype or florid cartilage invasion reported in other cases, but tracheal staining patterns for GAGs for our patient were very similar to those seen in normal trachea using established protocols for mucopolysaccharidosis histopathology [12]. Increases in chondrocyte lacunar size certainly warrant corroboration and further investigation in this patient group as they would be consistent with the clinical picture of tracheal tissue laxity. Given the comparable LAMP1 staining and overall GAG staining to normal trachea and the lack of macrophage numbers or phenotypes consistent with active cartilage breakdown, the increase in chondrocyte lacunae size would be more consistent with a slowed but still abnormal cartilage breakdown and removal following ERT commencement, causing a less rigid cartilage structure due to decreased cartilage density. This suggests that, in this patient who had developed multilevel tracheal tortuosity despite having been on ERT for a decade, the driving forces for his tracheal defect were perhaps simple sequelae of his skeletal thoracic cage deformities on his less rigid tracheal cartilage. As our patient series grows in number, more meaningful analysis will be able to be undertaken to ascertain the underlying cause. Although it is unclear whether GAG accumulation represents a barrier to long-term outcomes, Morquio A syndrome itself was not a barrier to satisfactory short-term airway healing in this single patient on ERT.

Although there have been other cases reported in the literature of successful use of tracheal resection to ameliorate Morquio A syndrome's airway manifestations [13–15], these have been via median sternotomy approaches necessitating cardiopulmonary bypass at considerable risk to the patient. Our novel use of a transcervical approach without cardiopulmonary bypass appears to have satisfactorily addressed the mismatched longitudinal growth of the trachea, whilst avoiding the morbidity associated with extracorporeal membrane oxygenation, sternotomy, or tracheostomy. More importantly, this resection technique resulted in extremely beneficial improvements in the patient's quality of life, facilitating return to his normal activities of daily life.

## 4. Conclusion

In this case, we have shown tracheal resection via a transcervical approach without the use of cardiopulmonary bypass to be safe and effective in significantly improving the burden of airway symptoms associated with Morquio A syndrome. In the right multidisciplinary and holistic setting, this could open the technique up as an option for other similarly affected patients in the future.

## Data Availability

No data were used to support the study.

## Abbreviations

BiPAP: Bilevel positive airway pressure
C6S: Chondroitin-6-sulphate
CD68: Glycosylated glycoprotein marker for macrophages
c-spine: Cervical spine
CT: Computerised tomography
ENT: Otolaryngology
ERT: Enzyme replacement therapy
ETT: Endotracheal tube
GAG: Glycosaminoglycan
GALNS: N-acetylgalactosamine-6-sulphate sulfatase
GBI: Glasgow Benefit Inventory
GOSH: Great Ormond Street Hospital
IMD: Inherited metabolic disorders
KS: Keratan sulphate
LAMP1: Lysosomal associated membrane protein 1
MFT: Manchester University NHS Foundation Trust
MPS: Mucopolysaccharidosis
NHS: National Health Service
NIV: Noninvasive ventilatory support
PDS: Polydioxanone
POD: Postoperative day.

## Additional Points

*Postprocedure Quality of Life Assessment*. Dynamic changes in quality of life, both before and after the procedure, were assessed using the Glasgow Benefit Inventory (GBI). The GBI is a validated postinterventional instrument that is appropriate for measuring changes in health status postotorhinolaryngology interventions, by employing generic patient-recorded outcome measure (PROM) [1]. This was first described by Robinson et al. (1996) comparing quality of life metrics before and after an intervention and has also been validated by Halliday et al. (2020) for indications beyond surgical otorhinolaryngology, in this case benefits of lymphoedema treatment in patients with head and neck cancer [2, 3]. Whilst the patient is an adolescent that is in a transitional milieu, the GBI has been validated for this age (i.e., the transitional age groups) and has been validated for contextualisation to novel otorhinolaryngological interventions, as demonstrated by Halliday et al. (2020) [3]. The GBI consists of a questionnaire of 18 questions, with each one centred on a five-point Likert rating scale (i.e., having a score of one to five). The questionnaires encompass health-related aspects of general, physical, and social health. The scores range from −100 to +100, with a score of zero indicating no benefit (or maximum harm) and a score of +100 indicating maximum benefit. For this case, the result of the GBI questionnaire was 77/100 with particularly strong scores attained for metrics assessing physical health and psychological well-being. Of note, maximum scores were achieved in areas addressing improvements in respiratory function, activities of daily living, and self-confidence, particularly when addressing potential future vocational prospects. The changes in quality of life are clearly illustrated from a narrative account provided by the parents (Figure 4).

## Supplementary Materials

Supplementary Materials Video 1: Preoperative microlaryngobronchoscopy for airway assessment. Video 2: Postoperative (day 7) microlaryngobronchoscopy for airway assessment. Link to supplementary video folder: https://drive.google.com/drive/folders/1_x2SvlmyhDaVMvv1JKA01Ba4Pe1xkQbE?usp=sharing.
